# *FOXP1* inhibits cell growth and attenuates tumorigenicity of neuroblastoma

**DOI:** 10.1186/1471-2407-14-840

**Published:** 2014-11-18

**Authors:** Sandra Ackermann, Hayriye Kocak, Barbara Hero, Volker Ehemann, Yvonne Kahlert, André Oberthuer, Frederik Roels, Jessica Theißen, Margarete Odenthal, Frank Berthold, Matthias Fischer

**Affiliations:** Department of Pediatric Oncology and Hematology and Center for Molecular Medicine Cologne (CMMC), Children’s Hospital, University of Cologne, Kerpener Straße 62, Cologne, 50924 Germany; Institute of Pathology (INF 220), University of Heidelberg, Heidelberg, 69120 Germany; Institute of Pathology, University of Cologne, Cologne, 50924 Germany

**Keywords:** FoxP1, Neuroblastoma, Tumor suppressor, Cell proliferation, Disease progression

## Abstract

**Background:**

Segmental genomic copy number alterations, such as loss of 11q or 3p and gain of 17q, are well established markers of poor outcome in neuroblastoma, and have been suggested to comprise tumor suppressor genes or oncogenes, respectively. The gene *forkhead box P1* (*FOXP1*) maps to chromosome 3p14.1, a tumor suppressor locus deleted in many human cancers including neuroblastoma. FoxP1 belongs to a family of winged-helix transcription factors that are involved in processes of cellular proliferation, differentiation and neoplastic transformation.

**Methods:**

Microarray expression profiles of 476 neuroblastoma specimens were generated and genes differentially expressed between favorable and unfavorable neuroblastoma were identified. *FOXP1* expression was correlated to clinical markers and patient outcome. To determine whether hypermethylation is involved in silencing of *FOXP1*, methylation analysis of the 5′ region of *FOXP1* in 47 neuroblastomas was performed. Furthermore, *FOXP1* was re-expressed in three neuroblastoma cell lines to study the effect of *FOXP1* on growth characteristics of neuroblastoma cells.

**Results:**

Low expression of *FOXP1* is associated with markers of unfavorable prognosis like stage 4, age >18 months and *MYCN* amplification and unfavorable gene expression-based classification (*P* < 0.001 each). Moreover, *FOXP1* expression predicts patient outcome accurately and independently from well-established prognostic markers. Array-based CGH analysis of 159 neuroblastomas revealed that heterozygous loss of the *FOXP1* locus was a rare event (n = 4), but if present, was associated with low *FOXP1* expression. By contrast, DNA methylation analysis in 47 neuroblastomas indicated that hypermethylation is not regularly involved in *FOXP1* gene silencing. Re-expression of FoxP1 significantly impaired cell proliferation, viability and colony formation in soft agar. Furthermore, induction of *FOXP1* expression led to cell cycle arrest and apoptotic cell death of neuroblastoma cells.

**Conclusions:**

Our results suggest that down-regulation of *FOXP1* expression is a common event in high-risk neuroblastoma pathogenesis and may contribute to tumor progression and unfavorable patient outcome.

**Electronic supplementary material:**

The online version of this article (doi:10.1186/1471-2407-14-840) contains supplementary material, which is available to authorized users.

## Background

Neuroblastoma is the most common extracranial solid cancer in childhood and the most common cancer in infancy [1]. The tumor is suggested to originate from immature neuroblasts giving rise to the sympathetic nervous system. The clinical hallmark of neuroblastoma is its heterogeneity, with the likelihood of cure varying widely between distinct patient subgroups [2, 3]. Like no other tumor entity, neuroblastoma provides unique features of tumor biology, including subgroups with differentiation into benign ganglioneuroma and a high incidence of complete spontaneous regression in the absence of any or with minimal therapeutic intervention [4]. By contrast, high-risk neuroblastoma patients have a particularly poor 5-year survival rate of <40% despite intensive multimodal therapy compared to an average survival rate of 80% among other pediatric malignancies [5]. The molecular mechanisms underlying these distinct neuroblastoma phenotypes are still poorly understood.

The winged helix transcription factor Forkhead box P1 (FoxP1) is one of four members of the subfamily P of the Fox transcription factor family, which is characterized by a 100-amino acid long, evolutionarily conserved DNA-binding domain called Forkhead or winged-helix domain. Members of the Fox subfamily P share several characteristics that are atypical among Fox proteins: their Forkhead domain is located near the carboxy-terminal region and they contain leucine zipper motifs that promote FoxP homo- and heterodimerization and thus highly selective, tissue- or cell-type specific activity [6]. These factors are widely but not ubiquitously expressed in human tissues and have been implicated in both embryonic development and adult tissue homeostasis by regulating cell growth, proliferation, differentiation, longevity and transformation [7, 8]. Targeted deletion of *FOXP1* is lethal in embryogenesis, which is mainly due to cardiac defects [9]. Loss of FoxP1 function has been reported in several human malignancies such as endometrial cancer, lung cancer, head and neck cancer, prostate cancer, renal cell carcinoma, ovarian carcinoma, and has been shown to be associated with poor prognosis in breast cancer [8, 10, 11]. Furthermore, genomic loss at the tumor suppressor locus 3p14.1 including the *FOXP1* gene has been described in many cancer types [12] including neuroblastoma [13] and has indicated the presence of a neuroblastoma-associated putative tumor suppressor gene located between 3p14.1 and 3p21.32 [14]. On the other hand, *FOXP1* may also act as an oncogene as it is highly expressed in hepatocellular carcinoma and certain B cell malignancies, in which it is frequently targeted by activating chromosome translocations placing it under the transcriptional control of the IGH enhancers [15, 16].

To elucidate the role of FoxP1 in neuroblastoma pathogenesis, we examined the expression of *FOXP1* in a cohort of 476 primary neuroblastomas using microarrays, and assessed its correlation with prognostic markers and patient outcome. To investigate the mechanisms of *FOXP1* downregulation, we determined copy number aberrations of the *FOXP1* locus in 159 neuroblastomas by array-CGH as well as DNA methylation patterns of the *FOXP1* promoter in 47 tumors using mass spectrometry. Furthermore, we characterized the functional consequences of *FOXP1* re-expression on cell proliferation, apoptosis, migration and colony formation in neuroblastoma cell lines.

## Methods

### Gene expression microarray data and patients’ characteristics

Single-color gene expression profiles were generated from 476 primary neuroblastoma samples (stage 1, n = 118; stage 2, n = 78; stage 3, n = 71; stage 4, n = 148; stage 4S, n = 61) and 3 neuroblastoma cell lines (IMR-32, CHP-212 and SK-N-BE(2)) using a 44 K oligonucleotide microarray as described elsewhere [17]. All patients were registered in respective clinical trials with written informed consent from the patient and/or a parent/legal guardian. We received ethics approval from the Ethics Commission of the Faculty of Medicine of Cologne University for the clinical trials NB97 (NCT00017225) and NB2004 (NCT00410631) including the molecular assessment of tumor material. *MYCN*-amplification was observed in 66 tumors, while it was absent in 405 tumors (not determined, n = 5). Patients’ age at diagnosis ranged from 0 to 296 months (median age, 13 months). Median follow-up for patients without events was 7.6 years (range, 0.4-19.0 years). Stage was classified according to the International Neuroblastoma Staging System (INSS) [18]. Total RNA of *FOXP1*- and *GFP*-expressing IMR-32, CHP-212 and SK-N-BE(2) cells was isolated at 0, 12, 24 and 72 h after transgene induction using Trizol (Life Technologies, Darmstadt, Germany). All raw and normalized microarray data are available as a subset at Gene Expression Omnibus (Accession numbers GSE45480 and GSE62419). To determine global differences in the expression profiles of FoxP1- and GFP-induced IMR-32, CHP-212 and SK-N-BE(2) cells, we compared mean expression levels of each gene between *FOXP1*-induced and control cells as described previously [19]. Differentially expressed genes, either up- or down-regulated after *FOXP1* re-expression were determined by applying a fold-change cutoff (≥2) and performing an unpaired, two-tailed Student’s *t*-test. The Gene Set Enrichment Analysis (GSEA) software (Broad Institute, Cambridge, MA, USA) was used to identify coordinated changes in a priori defined sets of functionally grouped genes as described previously [20, 21]. The neuroblastoma subgroups used for methylation and array-CGH analyses consisted of 47 and 159 tumors, respectively. Risk estimation of corresponding patients was performed according to the International Neuroblastoma Risk Group (INRG) classification system (methylation analysis: high risk, n = 22; intermediate and low risk, n = 25; array-CGH analysis: high risk, n = 51; intermediate and low risk, n = 108) [22].

### Analysis of genomic aberrations and promoter methylation of *FOXP1*

Array-CGH profiles from 159 neuroblastoma tumors were generated using 44 K or 105 K oligonucleotide microarrays as described previously [23, 24]. The raw data were analyzed by Agilent Genomics Workbench software (v. 7.0; Agilent Technologies, Santa Clara, CA, USA, 2012) [23]. Array-CGH data are available as a subset at Gene Expression Omnibus (Accession: GSE45480).

*FOXP1* promoter methylation analysis was performed by Sequenom Inc. (Hamburg, Germany) as described elsewhere [19, 25]. Genomic DNA from 47 primary neuroblastoma specimens (high *FOXP1* expression, n = 23; low *FOXP1* expression, n = 24, as defined by the cutoff value for dichotomization of *FOXP1* expression) and the neuroblastoma cell line IMR-32 was used to sequence three selected DNA regions covering 45 CpG units downstream of the *FOXP1* start site (Table 1). PCR Primers were designed by using Methprimer (http://www.urogene.org/methprimer/, Additional file 1: Table S1).

**Table 1:** **Design statistics of genomic regions analyzed for methylation**

Number of DNA samples	48
Number of genomic regions	1
Number of amplicons	3
Number of CpG units	45
Median amplicon length	413 bp (min = 365; max = 435)
Median CpG/amplicon	14 CpG/amplicon (min = 11; max = 20)

### Data analysis and statistics

Statistical analysis was performed using SPSS software version 20.0 (IBM). Levels of *FOXP1* mRNA determined by microarray analysis were compared in patient groups defined by *MYCN* status (normal *vs*. amplified), age (<18 months *vs*. >18 months), tumor stage (stage 1–3 *vs*. 4S *vs*. stage 4) and gene expression-based classification (favorable *vs*. unfavorable) according to the published PAM classifier [26]. Two-tailed nonparametric tests (Wilcoxon, Mann–Whitney U, and Kruskal–Wallis test) were used where appropriate. Kaplan-Meier estimates for OS and EFS were calculated and compared by log-rank test. Recurrence, progression and death from disease were considered as events. The cutoff value for dichotomization of *FOXP1* expression was estimated by maximally selected log-rank statistics [27]. Multivariate analysis was performed for EFS and OS using Cox’s proportional hazards regression models. The factors *FOXP1* (low *vs*. high as reference), INSS stage (4 *vs*. 1, 2, 3, 4S as reference), *MYCN* (amplified *vs*. normal as reference) and age at diagnosis (≥18 months *vs*. <18 months as reference) were fitted into a backward selection. The criterion for inclusion was a likelihood-ratio test p-value less than 0.05 and for exclusion more than 0.10. Quantitative data for functional analyses are shown as means ± SD. Unpaired two-tailed Students t-tests were used where appropriate.

### Cell culture

The neuroblastoma cell lines SK-N-BE (2) and CHP-212 were obtained from American Tissue Culture Collection (ATCC, Rockville, MD, USA) and authenticated at the DSMZ-German Collection of Microorganisms and Cell Cultures (Braunschweig, Germany). The neuroblastoma cell line IMR-32 was purchased from the DSMZ and PT67 retroviral packaging cells were obtained from Clontech (Mountain View, CA). All parental and inducible cell lines were cultured and maintained as described previously [19]. Doxycycline (Sigma-Aldrich, Deisenhofen, Germany) was used to induce transgene expression (2 μg/ml).

### Plasmids and retroviruses

A human *FOXP1* full open reading frame cDNA clone (pCMV6-XL5-FOXP1, clone ID SC103781) was obtained from Origene (Rockville, MD) and cloned into the pRevTRE vector (Clontech) as described elsewhere [19]. PCR primer sequences were as follows: 5′-GAGGATCCACCATGATGCAAGAATCTGGGACTGAGACAAAG-3′ (forward) and 5′-CACAAGCTTTCACTCCATGTCCTCGTTTACTGGTTC-3′ (reverse), containing restriction sites for *BamH* I and *Hind* III, respectively. The control plasmid pRevTRE-eGFP-PRE has been described previously [19].

### Stable inducible neuroblastoma cell lines

Neuroblastoma cell lines stably expressing either *FOXP1* or *GFP* under the control of the reverse tetracycline-controlled transactivator (rtTA) were generated using the RevTet^™^ System (Clontech) as described previously [19].

### Western blot analysis

Immunoblots were prepared as described previously [28]. FoxP1 protein levels were analyzed 48 h after induction of transgene expression using primary mouse anti-FOXP1 antibody (dilution 1:1000; ab16645; Abcam, Cambridge, MA) and horseradish peroxidase-labeled secondary polyclonal goat anti-mouse antibody (dilution 1:1000; P0447, Dako, Glostrup Denmark). The antigen-antibody complex was detected with Visualizer Spray & Glow (Upstate, Schwalbach, Germany).

### *In vitro*growth properties assays

The effect of *FOXP1* expression on cell viability was assessed using the Trypan Blue dye exclusion test. Neuroblastoma cells were seeded in 48-well plates at a density of 1 × 10^4^ cells/well in 200 μl RPMI-1640 (10% Tet-free FCS, 2 μg/ml doxycycline). To ensure continuous supply of nutrients throughout the measurement interval, 400 μl fresh RPMI-1640 (10% Tet-free FCS, 2 μg/ml doxycycline) per well were added on day 4. Cells were harvested at day 6 and evaluated for cell number and viability by Trypan Blue exclusion. The effect of *FOXP1* expression on cell proliferation was assessed using the Alamar Blue assay (Life Technologies) according to the manufacturer’s instructions. In brief, neuroblastoma cells were seeded in 48-well plates at a density of 6 × 10^3^ cells/well in 200 μl RPMI-1640 (10% Tet-free FCS, 2 μg/ml doxycycline). To ensure continuous supply of nutrients throughout the measurement interval, 400 μl fresh RPMI-1640 (10% Tet-free FCS, 2 μg/ml doxycycline) per well were added on day 4. At day 6, 60 μl of Alamar Blue solution were added to each well, plates were incubated for 6 h and absorbance was measured at 570 nm against a reference wavelength of 595 nm using a plate reader (Multiscan Ascent plate reader; Thermo Labsystems, Helsinki, Finland). The relative reduction of Alamar Blue was calculated as described by the manufacturer. Cell cycle distribution was assessed by FACS analysis as described previously [29].

### Analysis of apoptosis

Annexin-V-binding analysis was carried out using APC-coupled Annexin-V and 7-AAD according to the manufacturer’s protocol (BD Biosciences, Heidelberg, Germany). Apoptotic cells were detected by FACS (FACS Canto; BD Biosciences) using the DIVA software (BD Biosciences). TUNEL analysis was performed 3 days after induction of *FOXP1* using the In Situ Cell Death Detection Kit according to the manufacturer’s protocol (Roche Diagnostics, Mannheim, Germany) as described previously [19].

### Colony formation assay

A soft agar assay was used to assess the effect of *FOXP1* on colony-forming of neuroblastoma cells as described previously [19]. After 15 days of incubation at 37°C, colonies were stained with 0.005% (w/v) crystal violet (Life Technologies) and colonies larger than 100 μm diameter were counted.

### Migration analysis

To assess the effect of *FOXP1* on neuroblastoma cell motility, two different assays were used: a transwell (Boyden chamber) assay and a wound healing assay. First, a Boyden chamber transwell migration assay was performed according to the manufacturer’s protocol. In brief, 500 μl RPMI-1640 (20% Tet-free FCS, 2 μg/ml doxycycline) were added in each lower chamber of a 24-well transwell plate (No. 3421, 5.0 μm pore size, Costar, Corning Costar, Rochester, NY), and 300 μl cell suspension (0.5 × 10^6^ cells/ml) of IMR-32 and SK-N-BE(2) *FOXP1* or *GFP* transgenic cells in RPMI-1640 (FCS-free, 2 μg/ml doxycycline) were seeded in the upper chamber. Cultures were maintained for 6 days, then non-motile cells at the top of the filter were removed and the cells on the lower surface of the membrane were fixed with methanol and stained with DAPI. The number of cells that had migrated to the lower surface of the membrane was counted in four random fields at 10 × 10 magnification under a light microscope.

As a second method to determine the effect of *FOXP1* re-expression on migration of neuroblastoma cells, CHP-212 and SK-N-BE(2) cells were analyzed in a wound healing assay using IBIDI culture inserts (No. 80209, IBIDI GmbH, Martinsried, Germany) according to the manufacturer’s protocol. An IBIDI culture insert consists of two reservoirs separated by a 500 mm thick wall. In brief, an IBIDI culture insert was placed into one well of the 24 well plate and slightly pressed on the top to ensure tight adhesion. Equal numbers of *FOXP1* or *GFP* transgenic cells (70 μl per reservoir; 1.0 × 10^5^ cells/ml) in RPMI-1640 (10% Tet-free FCS) were added into the inserts. After 24 hours, the insert was gently removed, creating a gap of 500 μm. The well was filled with RPMI-1640 (10% Tet-free FCS, 2 μg/ml doxycycline) and the migration was documented at 10 × 10 magnification under a light microscope.

All assays were conducted in triplicate and reproduced in two independent experiments.

## Results

### Low *FOXP1*transcript levels are correlated with markers of poor outcome in neuroblastoma

**Figure 1:**
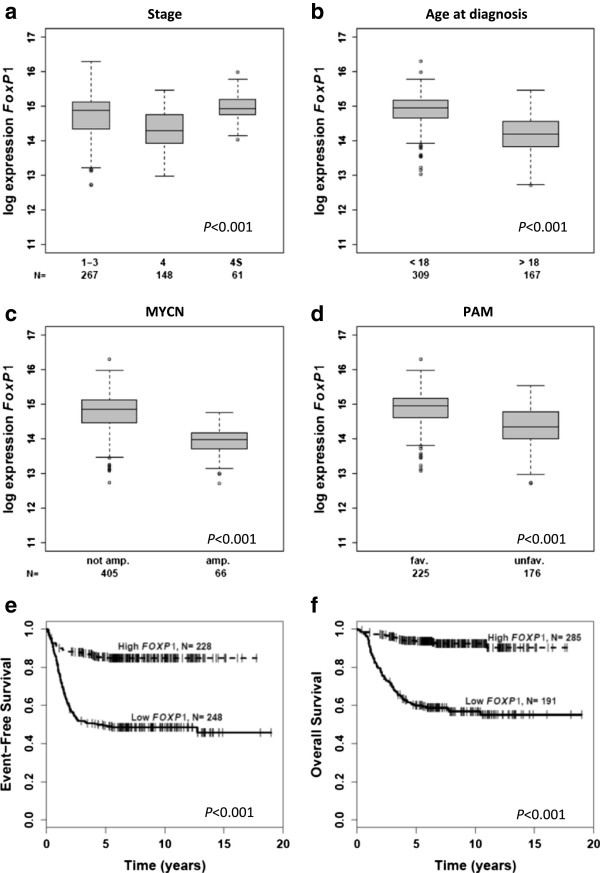
**Association of** ***FOXP1*** **expression levels with prognostic markers and patient outcome in 476 neuroblastomas as determined by microarray analysis.** Association of **(a)** tumor stage, **(b)** age at diagnosis, **(c)** *MYCN* amplification status and **(d)** gene expression-based classification (PAM classifier) [26] with *FOXP1* transcript levels. Boxes, median expression values (horizontal line) and 25th and 75th percentiles; whiskers, distances from the end of the box to the largest and smallest observed values that are less than 1.5 box lengths from either end of the box; open circles, outlying values. Survival curves of neuroblastoma patients for **(e)** EFS and **(f)** OS according to *FOXP1* expression as determined by microarray analysis. N, patient numbers; amp, amplified; fav, favorable; unfav, unfavorable.

To investigate whether *FOXP1* expression is associated with prognostic markers of poor outcome in neuroblastoma, its expression levels were evaluated in a cohort of 476 neuroblastoma microarray profiles reflecting the whole spectrum of the disease [17]. We observed similar transcript levels in localized and stage 4S tumors, while expression levels were significantly lower in stage 4 neuroblastoma (Figure 1a; *P* < 0.001). *FOXP1* levels were substantially decreased in tumors of patients older than 18 months at diagnosis (Figure 1b; *P* < 0.001) as well as in tumors with *MYCN* amplification (Figure 1c; *P* < 0.001). Furthermore, low *FOXP1* transcript levels significantly correlated with unfavorable gene expression-based classification (Figure 1d; *P* < 0.001) according to a prognostic multigene classifier that we have defined previously [26]. This predictive signature consists of 144 genes, but does not include *FOXP1*. Taken together, these results demonstrate that downregulation of *FOXP1* is associated with unfavorable prognostic markers in neuroblastoma.

### Low *FOXP1*expression is associated with adverse outcome of neuroblastoma patients

We next determined whether *FOXP1* expression is associated with neuroblastoma patient event-free survival (EFS) and overall survival (OS). We noticed that low *FOXP1* expression was associated with poor EFS (Figure 1e; 5-year EFS 0.49 ± 0.03 *vs*. 0.85 ± 0.02; *P <* 0.001) and OS (Figure 1f; 5-year OS 0.60 ± 0.04 *vs*. 0.93 ± 0.02; *P <* 0.001), demonstrating that *FOXP1* transcript levels discriminate neuroblastoma patients with beneficial and adverse outcome. The prognostic value of *FOXP1* expression was substantiated by multivariate Cox regression models based on EFS and OS considering prognostic markers that are currently used for neuroblastoma risk stratification. In these analyses, *FOXP1* expression turned out to be a significant independent prognostic marker for both EFS and OS (Table 2; EFS, hazard ratio, 2.29, 95% confidence interval, 1.46 – 3.58, *P* < 0.001; OS, hazard ratio, 1.79, 95% confidence interval, 0.98 – 3.26, *P* < 0.001).

**Table 2:** **Multivariate Cox regression models based on EFS and OS, considering single prognostic markers and** ***FOXP1*** **expression**

Marker	Patients (N)	Available cases (N)	Hazard ratio	95% CI	P-value
*Model considering single prognostic markers and FOXP1 expression based on EFS*	*476*	*471*			
Age (<18 months vs. >18 months)			1.43	0.96-2.13	0.077
Stage (stage 1-3, 4S vs. 4)			2.31	1.62-3.31	< 0.001
*MYCN* status (normal vs. amplified)			1.74	1.20-2.52	0.005
*FOXP1* expression (cutoff EFS ≥27 840 vs. <27 840)			2.29	1.46-3.58	< 0.001
*Model considering single prognostic markers and FOXP1 expression based on OS*	*476*	*471*			
Age (<18 months vs. >18 months)			2.95	1.60-5.42	< 0.001
Stage (stage 1-3, 4S vs. 4)			3.18	1.95-5.21	< 0.001
*MYCN* status (normal vs. amplified)			2.89	1.86-4.51	< 0.001
*FOXP1* expression (cutoff OS ≥26 428 vs. <26 428)			1.79	0.98-3.26	< 0.001

*Abbreviations: CI* confidence interval, *EFS* event-free survival, *OS* overall surviaval, *vs*. versus.

### Genetic and epigenetic characterization of the *FOXP1*locus in neuroblastoma

We next aimed to evaluate whether deregulated *FOXP1* expression is associated with genetic or epigenetic aberrations of the *FOXP1* locus.

**Figure 2:**
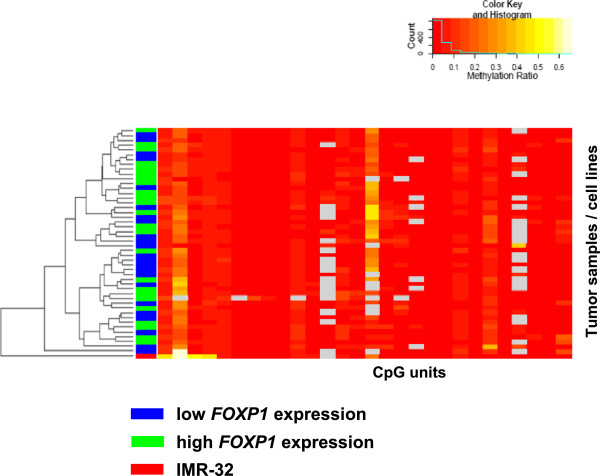
**Hierarchical clustering of** ***FOXP1*** **DNA methylation ratios.** A total of 45 CpG units of *FOXP1* were analyzed in 47 tumor samples and the neuroblastoma cell line IMR-32 (indicated in red). *FOXP1* expression levels of the tumors (blue, low; green, high; as defined by the expression cutoff value for dichotomizing patient EFS) are indicated aside. DNA methylation values are indicated by colors ranging from dark red (non-methylated) to bright yellow (65% methylated). Poor quality data are indicated in grey. A histogram is given in the inset that indicates the frequency of each color in the hierarchical clustering.

First, we examined the methylation status of three DNA regions covering 45 CpG units downstream of the *FOXP1* start site in 47 primary neuroblastoma samples and the neuroblastoma cell line IMR-32, which expresses *FOXP1* at low levels endogenously. Unsupervised one-way hierarchical clustering revealed a low degree of methylation in general, and a largely homogeneous methylation pattern in tumors of both subgroups (low expression of *FOXP1 vs*. high expression of *FOXP1* as defined in Methods) and IMR-32 cells (Figure 2). These results suggest that aberrant DNA methylation is not regularly involved in *FOXP1* gene silencing in neuroblastoma.

**Figure 3:**
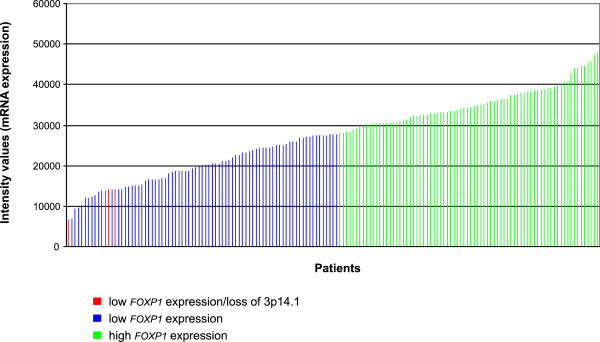
***FOXP1*** **mRNA expression intensities of 159 tumors for which array-CGH data were available.** Green and blue represent high and low *FOXP1* expression as defined by the expression cutoff value for dichotomizing patient EFS. Tumors showing loss of the *FOXP1* locus at 3p14.1 are indicated in red.

Next, we analyzed array-comparative genomic hybridization (array-CGH) profiles of 159 neuroblastoma samples and compared the results with corresponding microarray gene expression data of the same tumors. In four samples, segmental loss of chromosome 3p14.1 comprising the *FOXP1* locus was detected, suggesting that deletion of *FOXP1* is not a frequent genetic event in neuroblastoma. However, loss of *FOXP1* was accompanied by low *FOXP1* expression levels in all four cases (Figure 3), indicating that low *FOXP1* expression may be result from a gene dosage effect in a subset of neuroblastoma.

### Doxycycline-inducible expression of *FOXP1*in neuroblastoma cell lines

**Figure 4:**
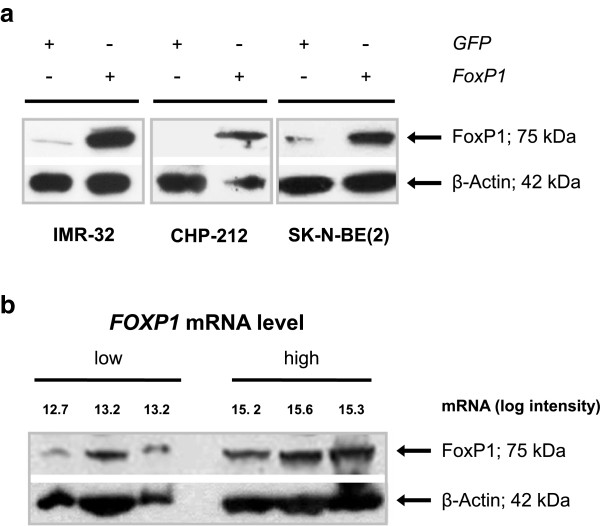
**Inducible expression of FoxP1 in neuroblastoma cell lines and physiological FoxP1 levels in primary neuroblastomas. (a)** Inducible FoxP1 protein expression in *FOXP1*- *versus GFP*-expressing neuroblastoma cell lines 48 h after induction of transgene expression. **(b)** Physiological FoxP1 protein levels in primary tumors; *FOXP1* transcript levels are indicated as log intensities (microarray data).

To examine *FOXP1* functions in neuroblastoma pathogenesis, we generated three stable, inducible *FOXP1* transgenic cell lines (IMR-32, CHP-212 and SK-N-BE(2)) using the retroviral RevTet-On^™^ System (Clontech). 48 h after induction of transgene expression, polyclonal FoxP1-expressing neuroblastoma cells were compared with polyclonal GFP-expressing cells due to promoter leakage of the pRevTRE Vector System (Figure 4a). Recombinant FoxP1 protein levels were in the range of physiological levels observed in neuroblastoma patients with high FoxP1 expression (Figure 4b). Notably, Western blot hybridization analysis revealed that *FOXP1* transcript levels correlate well with FoxP1 protein levels (Figure 4b), which may indicate that measurement of *FOXP1* mRNA is predictive of its functional activity in neuroblastoma.

### *FOXP1*expression inhibits neuroblastoma cell growth

**Figure 5:**
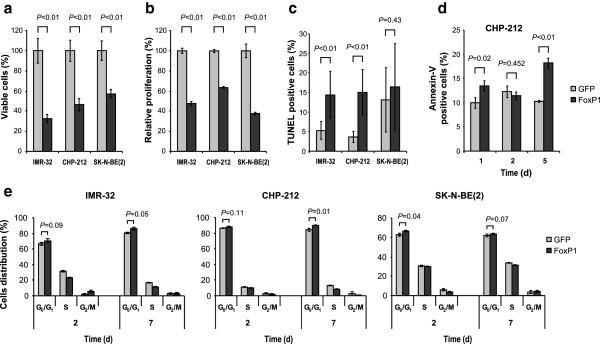
***FOXP1*** **re-expression inhibits growth of neuroblastoma cells.** FoxP1-induced changes in **(a)** cell viability (Trypan Blue dye exclusion analysis 6 days after induction of transgene expression), **(b)** cell proliferation (Alamar Blue assay 6 days after induction of transgene expression), **(c)** the proportion of cells showing apoptotic nuclei (72 h after induction of transgene expression), **(d)** the proportion of Annexin-V-positive cells (fluorescence-activated cell sorting (FACS)) and **(e)** cell cycle distribution (FACS). Each value represents the mean of triplicate experiments; error bars indicate S.D.

To investigate whether *FOXP1* expression is involved in the regulation of growth properties in neuroblastoma cells, *FOXP1*-expressing IMR-32, CHP-212 and SK-N-BE(2) cells were compared with *GFP*-expressing controls. Cell proliferation was assayed for 6 days using Trypan Blue dye exclusion tests and Alamar Blue assays. On day 6, the number of viable cells was significantly decreased in all *FOXP1*-expressing cell lines as compared to *GFP*-expressing controls (Figure 5a and b; *P* < 0.01).

We next examined whether apoptosis may contribute to the inhibition of neuroblastoma cell growth after *FOXP1* re-expression. First, DNA fragmentation was assessed using the terminal deoxynucleotidyl transferase-mediated dUTP nick-end labeling (TUNEL) assay in IMR-32, CHP-212 and SK-N-BE(2) cells. In IMR-32 and CHP-212, *FOXP1*-expressing cells showed a significantly higher fraction of TUNEL-positive cells as compared to *GFP*-expressing controls, while no difference in the frequency of apoptotic events was observed in SK-N-BE(2) cells (Figure 5c). Second, we analyzed the externalization of phosphatidylserine by flow cytometry using Annexin-V staining in CHP-212 cells. In accordance with the results of the TUNEL analysis, we observed a significant increase of the Annexin-V-binding fraction 5 days after *FOXP1* induction in comparison to control cells (Figure 5d).

To assess whether reduced proliferation upon *FOXP1* re-expression might be a consequence of impaired cell cycle progression, cell cycle distribution was determined by fluorescence-activated cell sorting (FACS) on days 2 and 7 after induction of *FOXP1* expression. We observed an increased fraction of cells in the G_0_/G_1_ phase at the expense of the S-phase population in *FOXP1*-expressing cells in all three cell lines (Figure 5e).

Taken together, these findings indicate that FoxP1 affects growth properties in human neuroblastoma cells by both cell cycle regulation and induction of apoptosis.

### *FOXP1*attenuates the malignant phenotype of neuroblastoma cells

**Figure 6:**
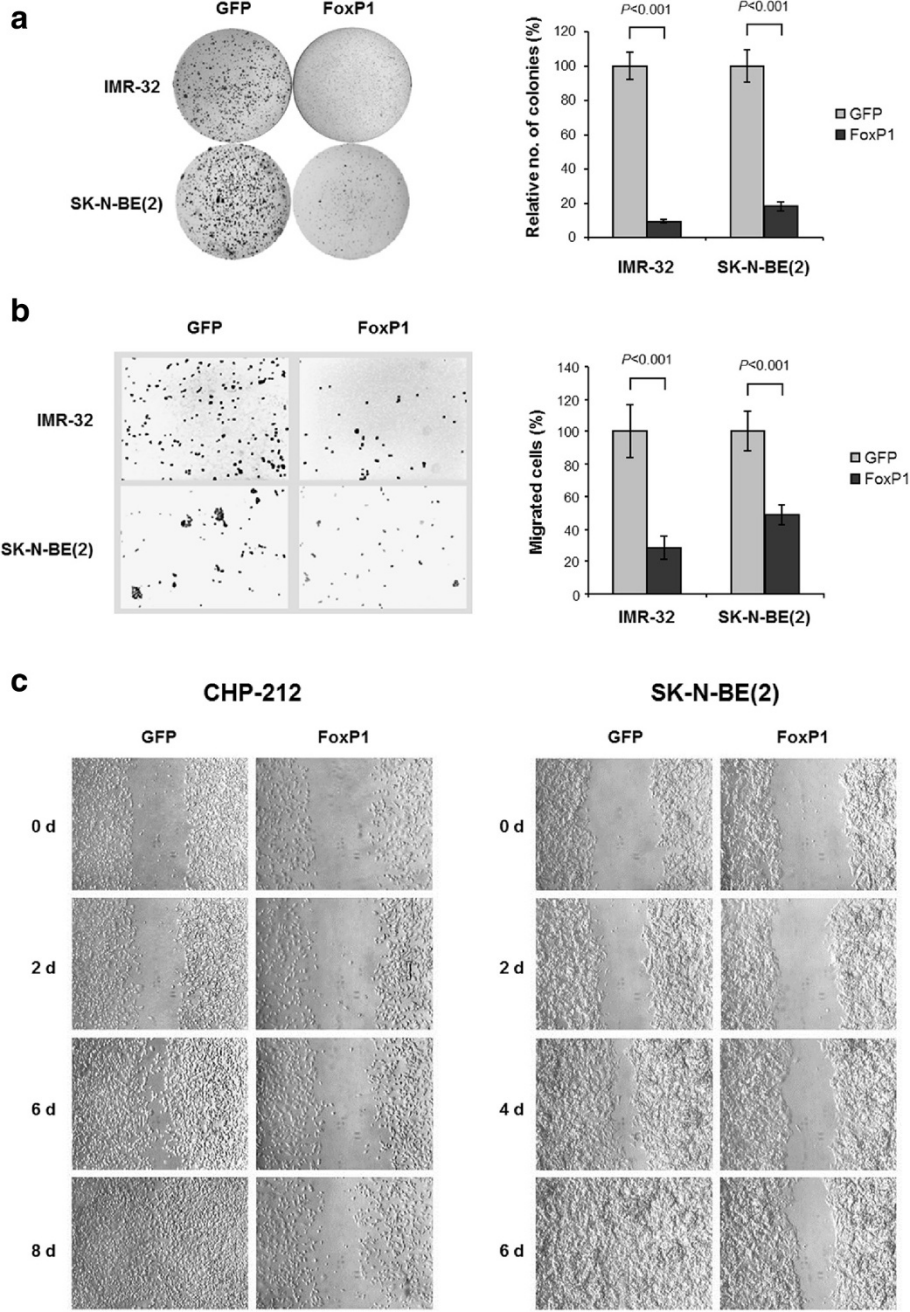
***FOXP1*** **attenuates the malignant phenotype of neuroblastoma cells. (a)** Soft agar colony formation assay of *FOXP1*- *versus GFP*-expressing control cells. Cells were cultured in soft agar for 15 days. **(b)** Boyden chamber migration assay of *FOXP1*- *versus GFP*-expressing control cells. Cells were seeded in Boyden chambers and incubated for 6 days. **(c)** Gap closure assay of *FOXP1*- *versus GFP*-expressing control cells. Images were captured at the indicated time points. Each value represents the mean of triplicate experiments; error bars indicate S.D. d, days; no., number.

To further assess the effect of *FOXP1* expression on tumorigenicity, we compared anchorage-independent growth rates and migration capabilities of *FOXP1*-induced with *GFP*-induced control cells. Fifteen days after transgene induction, *FOXP1*-expressing IMR-32 and SK-N-BE(2) cells showed a significantly reduced colony-forming ability in soft agar to about 10 and 20% of *GFP*-expressing control cells, respectively (Figure 6a; *P* < 0.001 each). In the Boyden chamber assay, the number of migrated cells in the lower chamber was significantly smaller in the *FOXP1* expressing IMR-32 and SK-N-BE(2) cells as compared to the *GFP*-expressing controls (Figure 6b; 30% and 50%, respectively; *P* < 0.001 each). Consistent with these results, gap closure was delayed in the *FOXP1*-induced CHP-212 and SK-N-BE(2) cells as compared to *GFP*-expressing controls (Figure 6c).

Together, our observations demonstrate that reconstitution of *FOXP1* expression suppresses the malignant phenotype of neuroblastoma cells.

### FoxP1 modulates the expression patterns of apoptosis-, migration- and differentiation-related genes

**Figure 7:**
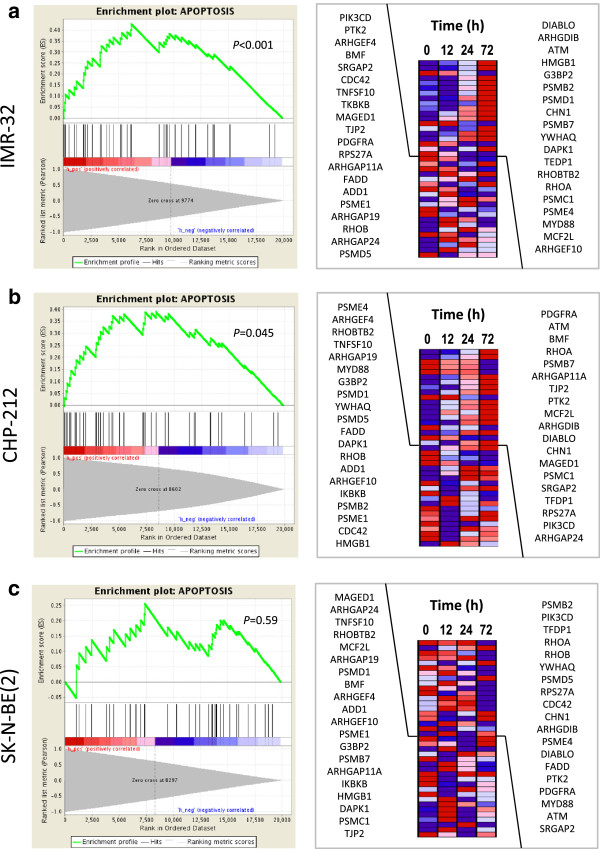
***FOXP1*** **induces genes involved in apoptosis.** Gene set enrichment analysis (GSEA) of time-resolved gene expression measurements in FoxP1-induced neuroblastoma cells. Cells were analyzed 0, 12, 24 and 72 hours after transgene induction. The enrichment score (ES) plotted as a function of the position within the ranked list of array probes is shown as a green line. The ranked list metric shown in gray illustrates the correlation of the gene expression values across the time series. Right, individual expression profiles for time leading edge probe sets contributing to the normalized enrichment score are shown. Signal intensities are illustrated by varying shades of red (up-regulation) and blue (down-regulation). FoxP1 up-regulates pro-apoptotic genes in **(a)** IMR-32 and **(b)** CHP-212 cells as compared to *GFP*-expressing controls. **(c)** Recombinant *FOXP1* fails to induce pro-apoptotic genes in the p53 mutant cell line SK-N-BE(2).

**Figure 8:**
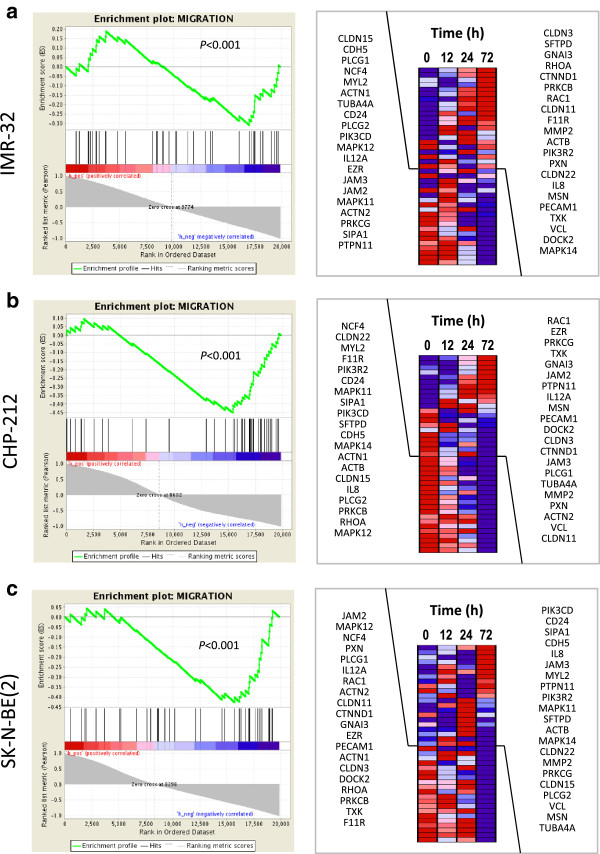
***FOXP1*** **down-regulates genes involved in migration.** Gene set enrichment analysis (GSEA) of time-resolved gene expression measurements in FoxP1-induced neuroblastoma cells. Cells were analyzed 0, 12, 24 and 72 hours after transgene induction. The enrichment score (ES) plotted as a function of the position within the ranked list of array probes is shown as a green line. The ranked list metric shown in gray illustrates the correlation of the gene expression values across the time series. Right, individual expression profiles for time leading edge probe sets contributing to the normalized enrichment score are shown. Signal intensities are illustrated by varying shades of red (up-regulation) and blue (down-regulation). FoxP1 decreases the expression of genes involved in migration processes in **(a)** IMR-32, **(b)** CHP-212 and **(c)** SK-N-BE (2) cells as compared to *GFP*-expressing controls.

To assess the molecular consequences of *FOXP1* re-expression in neuroblastoma cells, we performed series of time-resolved expression microarray analyses of *FOXP1*- and *GFP*-expressing IMR-32, CHP-212 and SK-N-BE(2) cells. Expression profiles were generated 0, 12, 24 and 72 hours after transgene induction. Gene set enrichment analysis (GSEA) revealed a significant induction of genes involved in apoptosis in IMR-32 (Figure 7a; enrichment score =0.43; *P* < 0.001) and CHP-212 (Figure 7b; enrichment score =0.39; *P* = 0.045) neuroblastoma cells. No enrichment was found in the p53 mutant cell line SK-N-BE(2) (Figure 7c; *P* = 0.59). The heatmap plots in Figure 7 show a set of 39 genes involved in apoptosis. Furthermore, all three model cell lines IMR-32 (Figure 8a; enrichment score = -0.31; *P* < 0.001), CHP-212 (Figure 8b; enrichment score = -0.45; *P* < 0.001) and SK-N-BE(2) (Figure 8c; enrichment score = -0.43; *P* < 0.001) showed a significant down-regulation of genes involved in cell migration. The heatmap plots in Figure 8 show a set of 41 genes involved in cell migration.

**Figure 9:**
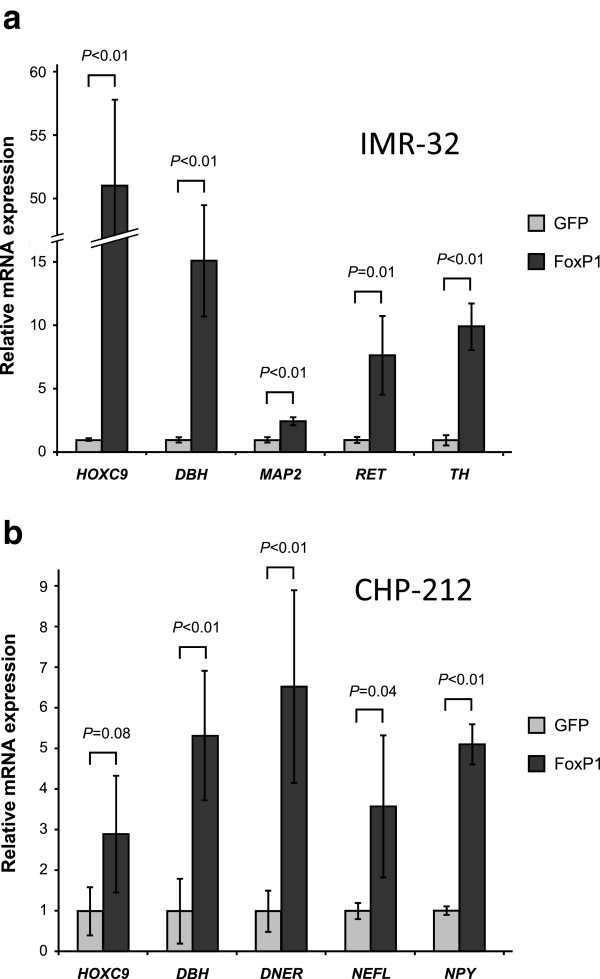
***FOXP1*** **induces genes involved in neuronal differentiation.** *FOXP1* expression up-regulates neuron-related markers in **(a)** IMR-32 and **(b)** CHP-212 cells as determined by microarray gene expression analysis. Error bars indicate S.D.

To identify downstream targets and signaling pathways regulated by *FOXP1*, we analyzed differentially expressed genes after *FOXP1* re-expression using microarray gene expression analysis (Additional file 2: Table S2). *FOXP1* strongly up-regulated the expression of the homeobox C9 gene (*HOXC9*) and genes involved in neuronal differentiation pathways such as *DBH*, *MAP2*, *RET*, *TH*, *DNER*, *NEFL* and *NPY* in IMR-32 (Figure 9a) and CHP-212 cells (Figure 9b). Induction of *HOXC9* or genes associated with neuronal differentiation was absent in the p53-mutated background of SK-N-BE(2) cells. By contrast, we found a strong (21.2-fold) induction of *TNFSF10* (*TRAIL*) in SK-N-BE(2) cells immediately after *FOXP1* induction (Figure 7c), indicating an alternative pathway of *FOXP1*-induced growth inhibition in SK-N-BE(2) cells.

## Discussion

Altered patterns of gene expression arising from inappropriate regulation or function of key transcription factors cause many human diseases, including cancer. Forkhead box proteins are attracting increasing attention as tissue type dependent regulators and critical mediators of cellular processes including proliferation, migration, differentiation, cell cycle arrest or cell death. Dysregulation of Fox protein expression has been implicated in a broad spectrum of human disorders, including immunological dysfunction, infertility, speech/language disorders, and malignancies [30, 31]. FoxP1 is a transcriptional repressor that plays an important role in the development of the brain and lung in mammals and has been described to have diverse and opposing functions in different types of cancer [32]. Results of recent studies indicate that loss of FoxP1 activity promotes solid tumor proliferation, e.g. in endometrial cancer, prostate cancer, renal cell carcinoma, and breast cancer, suggesting a tumor-suppressive role in these malignancies [16, 33]. In contrast, high expression of *FOXP1* was shown to correlate with poor outcome in certain types of B cell lymphomas [34–36]. While several Fox family members have been shown to play critical roles in neuroblastoma biology [37–40], the role of FoxP1 has not yet been investigated in this context.

Here, we demonstrate that low *FOXP1* expression is associated with unfavorable prognostic markers and poor patient outcome in neuroblastoma. The prognostic relevance of low *FOXP1* expression was validated by multivariate analysis for OS and EFS, showing that *FOXP1* expression predicts neuroblastoma patient outcome independently from well-established prognostic markers. Similar results have recently been published for prostate cancer and non-small cell lung cancer, in which an inverse correlation of *FOXP1* expression with increased malignancy grade and reduced patient survival was observed [33]. Together, these findings may suggest a tumor suppressive role of FoxP1 in neuroblastoma.

In line with this hypothesis, restoration of *FOXP1* expression strongly decreased growth rates and tumorigenic characteristics of all three neuroblastoma cell lines analyzed *in vitro*. These results are consistent with recent studies of other solid tumors, in which ectopic FoxP1 exhibited strong tumor suppressor activity in prostate cancer and glioma cells [33, 41]. Although the results of the migration analyses may in part be explained by a reduction in cell number rather than a change of cell migration ability, the analysis of mRNA levels confirmed down-regulation of cell motility-associated genes such as *MAPK12* [42], *PLCG2* [43] and *NCF4* [44] in all three *FOXP1* transgenic neuroblastoma cell lines analyzed. Considering that the cell lines used in this study differ in several molecular and biological characteristics like genetic alterations, doubling time, morphology and motility, our results may suggest that diminished *FOXP1* expression might act as a growth-promoting factor in neuroblastoma in general. We also found that induction of FoxP1 delayed cell cycle progression and up-regulated pro-apoptotic genes such as *DIABLO* [45], *CDC42* [46] and *DAPK1* [47], indicating the induction of the intrinsic pathway of apoptosis. In addition, we found that *HOXC9* is up-regulated, a well described key regulator of neuroblastoma differentiation which links the intrinsic pathway of apoptosis, cell-cycle exit and neuronal differentiation [48]. In line with previously published analyses of *HOXC9* in neuroblastoma cells, we found a significant up-regulation of genes involved in neuronal differentiation pathways such as *DNER*, *NEFL* [19], *DBH*, *TH* [48], *RET* [49], *MAP2* [50] and *NPY* [51]. The combined effects of intrinsic apoptosis and induction of differentiation have been described to strongly inhibit of proliferation and reduce tumorigenicity of neuroblastoma cells previously, which is well in line with the effects *FOXP1* re-expression shown here. Together, these results indicate that FoxP1 may mediate cellular growth restriction in neuroblastoma by cell cycle control, programmed cell death and induction of differentiation. Interestingly, recombinant FoxP1 failed to induce apoptosis in the p53 mutant cell line SK-N-BE(2), suggesting a p53 dependent mechanism of FoxP1 mediated cell death. In accordance with this finding, we did not observe an induction of *HOXC9* or other pro-apoptotic genes in this model cell line. FoxP1 and p53 signaling have been described to be connected in B-cell lymphoma, in which the oncogenic action of FoxP1 is repressed by p53-induced miR-34a [52]. These findings emphasize the need for further studies to elucidate the interaction of these factors and their signaling partners in a tissue-specific context. Of note, SK-N-BE(2) cells showed a strong (21.2-fold) induction of *TNFSF10*, the tumor necrosis factor-related apoptosis-inducing ligand (*TRAIL*), immediately after *FOXP1* induction (Figure 7c). This gene has been described to inhibit cell proliferation in several other tumor entities by regulating autophagy and extrinsic apoptosis, indicating an alternative pathway of *FOXP1*-induced growth inhibition in SK-N-BE(2) cells.

We also aimed to identify the molecular basis underlying the differential expression of *FOXP1* observed in different neuroblastoma subgroups. Loss of chromosome 3p is a genomic aberration frequently found in unfavorable neuroblastoma, suggesting that one or multiple tumor suppressor genes are targeted by this genetic alteration [13, 53–55]. We found low *FOXP1* expression in four tumor samples with segmental 3p14.1 loss containing the *FOXP1* locus, pointing towards haploinsufficiency in these tumors. However, deletions of the *FOXP1* locus appear to occur rarely in neuroblastoma, suggesting that other factors contribute to differential *FOXP1* expression in this malignancy. Analysis of DNA methylation patterns and *FOXP1* expression levels did not reveal any correlation, implicating that differential methylation is not a common cause of *FOXP1* repression in neuroblastoma. In addition, somatic mutations of the coding sequence of *FOXP1* have not been identified in recent reports on the mutational spectrum of neuroblastoma using next generation sequencing [56, 57]. We therefore speculate that additional molecular mechanisms may mediate *FOXP1* gene silencing in neuroblastoma. Aberrant expression of *FOXP1* in various pathologic conditions has been reported to be caused by a variety of molecular mechanisms, including dysregulation of upstream signaling pathways such as FoxO proteins or components of the estrogen-ER signaling pathway [58–60], differential splicing [61, 62], viral integration events [63], differential miRNA activity [64, 65] and translocations affecting upstream regulatory regions of the *FOXP1* gene [66]. Further investigations are required to determine the contribution of these mechanisms to altered *FOXP1* expression in neuroblastoma.

## Conclusions

In summary, our study indicates that aggressive human neuroblastomas show reduced *FOXP1* expression, a finding that is compatible with the previously observed loss of FoxP1 in several types of solid tumors. *FOXP1* expression levels are inversely correlated with proliferative activity and tumorigenicity *in vitro*, supporting its role as a transcriptional regulator with critical tumor suppressor functions in solid tumor biology. Although the mechanisms that regulate *FOXP1* expression in neuroblastoma remain unclear, low expression of *FOXP1* may add predictive value on top of established risk stratification tools in neuroblastoma. Further analysis of this transcription factor may contribute to the understanding of the molecular processes underlying tumor regression and progression in neuroblastoma.

## Electronic supplementary material

Additional file 1: Table S1: Information on oligonucleotides used as primers for DNA methylation analysis. (XLS 38 KB)

Additional file 2: Table S2: Genes up- or down-regulated after *FOXP1* re-expression in IMR-32, CHP-212 and SK-N-BE(2) cells as determined by microarray gene expression analysis. (XLSX 438 KB)

## References

[1] Schwab M, Westermann F, Hero B, Berthold F (2003). Neuroblastoma: biology and molecular and chromosomal pathology. Lancet Oncol.

[2] Coldman AJ, Fryer CJ, Elwood JM, Sonley MJ (1980). Neuroblastoma: influence of age at diagnosis, stage, tumor site, and sex on prognosis. Cancer.

[3] Maris JM, Hogarty MD, Bagatell R, Cohn SL (2007). Neuroblastoma. Lancet.

[4] Davidoff AM (2012). Neuroblastoma. Semin Pediatr Surg.

[5] Smith MA, Seibel NL, Altekruse SF, Ries LA, Melbert DL, O’Leary M, Smith FO, Reaman GH (2010). Outcomes for children and adolescents with cancer: challenges for the twenty-first century. J Clin Oncol.

[6] Lam EW, Brosens JJ, Gomes AR, Koo CY (2013). Forkhead box proteins: tuning forks for transcriptional harmony. Nat Rev Cancer.

[7] Kaufmann E, Knochel W (1996). Five years on the wings of fork head. Mech Dev.

[8] Koon HB, Ippolito GC, Banham AH, Tucker PW (2007). FOXP1: a potential therapeutic target in cancer. Expert Opin Ther Targets.

[9] Wang B, Weidenfeld J, Lu MM, Maika S, Kuziel WA, Morrisey EE, Tucker PW (2004). Foxp1 regulates cardiac outflow tract, endocardial cushion morphogenesis and myocyte proliferation and maturation. Development.

[10] Bates GJ, Fox SB, Han C, Launchbury R, Leek RD, Harris AL, Banham AH (2008). Expression of the forkhead transcription factor FOXP1 is associated with that of estrogen receptor-beta in primary invasive breast carcinomas. Breast Cancer Res Treat.

[11] Kim YS, Do Hwan J, Bae S, Bae DH, Ahn Shick W (2010). Identification of differentially expressed genes using an annealing control primer system in stage III serous ovarian carcinoma. BMC Cancer.

[12] Bieche I, Lidereau R (1995). Genetic alterations in breast cancer. Genes Chromosomes Cancer.

[13] Vandesompele J, Speleman F, Van Roy N, Laureys G, Brinskchmidt C, Christiansen H, Lampert F, Lastowska M, Bown N, Pearson A, Nicholson JC, Ross F, Combaret V, Delattre O, Feuerstein BG, Plantaz D (2001). Multicentre analysis of patterns of DNA gains and losses in 204 neuroblastoma tumors: how many genetic subgroups are there?. Med Pediatr Oncol.

[14] Scaruffi P, Coco S, Cifuentes F, Albino D, Nair M, Defferrari R, Mazzocco K, Tonini GP (2007). Identification and characterization of DNA imbalances in neuroblastoma by high-resolution oligonucleotide array comparative genomic hybridization. Cancer Genet Cytogenet.

[15] Espinet B, Garcia-Herrera A, Gallardo F, Baro C, Salgado R, Servitje O, Estrach T, Colomo L, Romagosa V, Barranco C, Serrano S, Campo E, Pujol RM, Sole F (2012). FOXP1 molecular cytogenetics and protein expression analyses in primary cutaneous large B cell lymphoma, leg-type. Histol Histopathol.

[16] Zhang Y, Zhang S, Wang X, Liu J, Yang L, He S, Chen L, Huang J (2012). Prognostic significance of FOXP1 as an oncogene in hepatocellular carcinoma. J Clin Pathol.

[17] Oberthuer A, Juraeva D, Li L, Kahlert Y, Westermann F, Eils R, Berthold F, Shi L, Wolfinger RD, Fischer M, Brors B (2010). Comparison of performance of one-color and two-color gene-expression analyses in predicting clinical endpoints of neuroblastoma patients. Pharmacogenomics J.

[18] Brodeur GM, Pritchard J, Berthold F, Carlsen NL, Castel V, Castelberry RP, De Bernardi B, Evans AE, Favrot M, Hedborg F, Kaneko M, Kemshead J, Lampert F, Lee REJ, Look T, Pearson ADJ, Philip T, Roald B, Sawada T, Seeger RC, Tsuchida Y, Voute PA (1993). Revisions of the international criteria for neuroblastoma diagnosis, staging, and response to treatment. J Clin Oncol.

[19] Kocak H, Ackermann S, Hero B, Kahlert Y, Oberthuer A, Juraeva D, Roels F, Theissen J, Westermann F, Deubzer H, Ehemann V, Brors B, Odenthal M, Berthold F, Fischer M (2013). Hox-C9 activates the intrinsic pathway of apoptosis and is associated with spontaneous regression in neuroblastoma. Cell Death Dis.

[20] Subramanian A, Tamayo P, Mootha VK, Mukherjee S, Ebert BL, Gillette MA, Paulovich A, Pomeroy SL, Golub TR, Lander ES, Mesirov JP (2005). Gene set enrichment analysis: a knowledge-based approach for interpreting genome-wide expression profiles. Proc Natl Acad Sci U S A.

[21] Prukop T, Epplen DB, Nientiedt T, Wichert SP, Fledrich R, Stassart RM, Rossner MJ, Edgar JM, Werner HB, Nave KA, Sereda MW (2014). Progesterone antagonist therapy in a Pelizaeus-Merzbacher mouse model. Am J Hum Genet.

[22] Cohn SL, Pearson AD, London WB, Monclair T, Ambros PF, Brodeur GM, Faldum A, Hero B, Iehara T, Machin D, Mosseri V, Simon T, Garaventa A, Castel V, Matthay KK (2009). The International Neuroblastoma Risk Group (INRG) classification system: an INRG Task Force report. J Clin Oncol.

[23] Fischer M, Bauer T, Oberthur A, Hero B, Theissen J, Ehrich M, Spitz R, Eils R, Westermann F, Brors B, Konig R, Berthold F (2009). Integrated genomic profiling identifies two distinct molecular subtypes with divergent outcome in neuroblastoma with loss of chromosome 11q. Oncogene.

[24] Spitz R, Oberthuer A, Zapatka M, Brors B, Hero B, Ernestus K, Oestreich J, Fischer M, Simon T, Berthold F (2006). Oligonucleotide array-based comparative genomic hybridization (aCGH) of 90 neuroblastomas reveals aberration patterns closely associated with relapse pattern and outcome. Genes Chromosomes Cancer.

[25] Ehrich M, Nelson MR, Stanssens P, Zabeau M, Liloglou T, Xinarianos G, Cantor CR, Field JK, van den Boom D (2005). Quantitative high-throughput analysis of DNA methylation patterns by base-specific cleavage and mass spectrometry. Proc Natl Acad Sci U S A.

[26] Oberthuer A, Berthold F, Warnat P, Hero B, Kahlert Y, Spitz R, Ernestus K, Konig R, Haas S, Eils R, Schwab M, Brors B, Westermann F, Fischer M (2006). Customized oligonucleotide microarray gene expression-based classification of neuroblastoma patients outperforms current clinical risk stratification. J Clin Oncol.

[27] Lausen B, Sauerbrei W, Schumacher M, Dirschedl P, Ostermann R (1994). Classification and regression trees (CART) used for the exploration of prognostic factors measured on different scales. Computational Statistics 25th Conference on Statistical Computing Edn.

[28] Ackermann S, Goeser F, Schulte JH, Schramm A, Ehemann V, Hero B, Eggert A, Berthold F, Fischer M (2011). Polo-like kinase 1 is a therapeutic target in high-risk neuroblastoma. Clin Cancer Res.

[29] Ehemann V, Hashemi B, Lange A, Otto HF (1999). Flow cytometric DNA analysis and chromosomal aberrations in malignant glioblastomas. Cancer Lett.

[30] Benayoun BA, Caburet S, Veitia RA (2011). Forkhead transcription factors: key players in health and disease. Trends Genet.

[31] Hannenhalli S, Kaestner KH (2009). The evolution of Fox genes and their role in development and disease. Nat Rev Genet.

[32] Tang B, Becanovic K, Desplats PA, Spencer B, Hill AM, Connolly C, Masliah E, Leavitt BR, Thomas EA (2012). Forkhead box protein p1 is a transcriptional repressor of immune signaling in the CNS: implications for transcriptional dysregulation in Huntington disease. Hum Mol Genet.

[33] Krohn A, Seidel A, Burkhardt L, Bachmann F, Mader M, Grupp K, Eichenauer T, Becker A, Adam M, Graefen M, Huland H, Kurtz S, Steurer S, Tsourlakis MC, Minner S, Michl U, Schlomm T, Sauter G, Simon R, Sirma H (2013). Recurrent deletion of 3p13 targets multiple tumor suppressor genes and defines a distinct subgroup of aggressive ERG fusion positive prostate cancers. J Pathol.

[34] Hoeller S, Schneider A, Haralambieva E, Dirnhofer S, Tzankov A (2010). FOXP1 protein overexpression is associated with inferior outcome in nodal diffuse large B-cell lymphomas with non-germinal centre phenotype, independent of gains and structural aberrations at 3p14.1. Histopathology.

[35] Lenz G, Wright GW, Emre NC, Kohlhammer H, Dave SS, Davis RE, Carty S, Lam LT, Shaffer AL, Xiao W, Powell J, Rosenwald A, Ott G, Muller-Hermelink HK, Gascoyne RD, Connors JM, Campo E, Jaffe ES, Delabie J, Smeland EB, Rimsza LM, Fisher RI, Weisenburger DD, Chan WC, Staudt LM (2008). Molecular subtypes of diffuse large B-cell lymphoma arise by distinct genetic pathways. Proc Natl Acad Sci U S A.

[36] Wlodarska I, Veyt E, De Paepe P, Vandenberghe P, Nooijen P, Theate I, Michaux L, Sagaert X, Marynen P, Hagemeijer A, De Wolf-Peeters C (2005). FOXP1, a gene highly expressed in a subset of diffuse large B-cell lymphoma, is recurrently targeted by genomic aberrations. Leukemia.

[37] Li D, Mei H, Qi M, Yang D, Zhao X, Xiang X, Pu J, Huang K, Zheng L, Tong Q (2013). FOXD3 is a novel tumor suppressor that affects growth, invasion, metastasis and angiogenesis of neuroblastoma. Oncotarget.

[38] Santo EE, Ebus ME, Koster J, Schulte JH, Lakeman A, van Sluis P, Vermeulen J, Gisselsson D, Ora I, Lindner S, Buckley PG, Stallings RL, Vandesompele J, Eggert A, Caron HN, Versteeg R, Molenaar JJ (2012). Oncogenic activation of FOXR1 by 11q23 intrachromosomal deletion-fusions in neuroblastoma. Oncogene.

[39] Wang Z, Park HJ, Carr JR, Chen YJ, Zheng Y, Li J, Tyner AL, Costa RH, Bagchi S, Raychaudhuri P (2011). FoxM1 in tumorigenicity of the neuroblastoma cells and renewal of the neural progenitors. Cancer Res.

[40] Obexer P, Hagenbuchner J, Unterkircher T, Sachsenmaier N, Seifarth C, Bock G, Porto V, Geiger K, Ausserlechner M (2009). Repression of BIRC5/survivin by FOXO3/FKHRL1 sensitizes human neuroblastoma cells to DNA damage-induced apoptosis. Mol Biol Cell.

[41] Xue L, Yue S, Zhang J (2014). FOXP1 has a low expression in human gliomas and its overexpression inhibits proliferation, invasion and migration of human glioma U251 cells. Mol Med Rep.

[42] Briet M, Schiffrin EL (2010). Aldosterone: effects on the kidney and cardiovascular system. Nat Rev Nephrol.

[43] Lamant L, de Reynies A, Duplantier MM, Rickman DS, Sabourdy F, Giuriato S, Brugieres L, Gaulard P, Espinos E, Delsol G (2007). Gene-expression profiling of systemic anaplastic large-cell lymphoma reveals differences based on ALK status and two distinct morphologic ALK + subtypes. Blood.

[44] Kim YM, Cho M (2014). Activation of NADPH oxidase subunit NCF4 induces ROS-mediated EMT signaling in HeLa cells. Cell Signal.

[45] Verhagen AM, Ekert PG, Pakusch M, Silke J, Connolly LM, Reid GE, Moritz RL, Simpson RJ, Vaux DL (2000). Identification of DIABLO, a mammalian protein that promotes apoptosis by binding to and antagonizing IAP proteins. Cell.

[46] Bazenet CE, Mota MA, Rubin LL (1998). The small GTP-binding protein Cdc42 is required for nerve growth factor withdrawal-induced neuronal death. Proc Natl Acad Sci U S A.

[47] Raval A, Tanner SM, Byrd JC, Angerman EB, Perko JD, Chen SS, Hackanson B, Grever MR, Lucas DM, Matkovic JJ, Lin TS, Kipps TJ, Murray F, Weisenburger D, Sanger W, Lynch J, Watson P, Jansen M, Yoshinaga Y, Rosenquist R, de Jong PJ, Coggill P, Beck S, Lynch H, de la Chapelle A, Plass C (2007). Downregulation of death-associated protein kinase 1 (DAPK1) in chronic lymphocytic leukemia. Cell.

[48] Mao L, Ding J, Zha Y, Yang L, McCarthy BA, King W, Cui H, Ding HF (2011). HOXC9 links cell-cycle exit and neuronal differentiation and is a prognostic marker in neuroblastoma. Cancer Res.

[49] Zha Y, Ding E, Yang L, Mao L, Wang X, McCarthy BA, Huang S, Ding HF (2012). Functional dissection of HOXD cluster genes in regulation of neuroblastoma cell proliferation and differentiation. PLoS One.

[50] Soltani MH, Pichardo R, Song Z, Sangha N, Camacho F, Satyamoorthy K, Sangueza OP, Setaluri V (2005). Microtubule-associated protein 2, a marker of neuronal differentiation, induces mitotic defects, inhibits growth of melanoma cells, and predicts metastatic potential of cutaneous melanoma. Am J Pathol.

[51] Andersson G, Pahlman S, Parrow V, Johansson I, Hammerling U (1994). Activation of the human NPY gene during neuroblastoma cell differentiation: induced transcriptional activities of AP-1 and AP-2. Cell Growth Differ.

[52] Craig VJ, Cogliatti SB, Imig J, Renner C, Neuenschwander S, Rehrauer H, Schlapbach R, Dirnhofer S, Tzankov A, Muller A (2011). Myc-mediated repression of microRNA-34a promotes high-grade transformation of B-cell lymphoma by dysregulation of FoxP1. Blood.

[53] Buckley PG, Alcock L, Bryan K, Bray I, Schulte JH, Schramm A, Eggert A, Mestdagh P, De Preter K, Vandesompele J, Speleman F, Stallings RL (2010). Chromosomal and microRNA expression patterns reveal biologically distinct subgroups of 11q- neuroblastoma. Clin Cancer Res.

[54] Scaruffi P, Stigliani S, Moretti S, Coco S, De Vecchi C, Valdora F, Garaventa A, Bonassi S, Tonini GP (2009). Transcribed-Ultra Conserved Region expression is associated with outcome in high-risk neuroblastoma. BMC Cancer.

[55] Spitz R, Hero B, Ernestus K, Berthold F (2003). Deletions in chromosome arms 3p and 11q are new prognostic markers in localized and 4 s neuroblastoma. Clin Cancer Res.

[56] Molenaar JJ, Koster J, Zwijnenburg DA, van Sluis P, Valentijn LJ, van der Ploeg I, Hamdi M, van Nes J, Westerman BA, van Arkel J, Ebus ME, Haneveld F, Lakeman A, Schild L, Molenaar P, Stroeken P, van Noesel MM, Ora I, Santo EE, Caron HN, Westerhout EM, Versteeg R (2012). Sequencing of neuroblastoma identifies chromothripsis and defects in neuritogenesis genes. Nature.

[57] Pugh TJ, Morozova O, Attiyeh EF, Asgharzadeh S, Wei JS, Auclair D, Carter SL, Cibulskis K, Hanna M, Kiezun A, Kim J, Lawrence MS, Lichenstein L, McKenna A, Pedamallu CS, Ramos AH, Shefler E, Sivachenko A, Sougnez C, Stewart C, Ally A, Birol I, Chiu R, Corbett RD, Hirst M, Jackman SD, Kamoh B, Khodabakshi AH, Krzywinski M, Lo A (2013). The genetic landscape of high-risk neuroblastoma. Nat Genet.

[58] Katoh M, Igarashi M, Fukuda H, Nakagama H (2013). Cancer genetics and genomics of human FOX family genes. Cancer Lett.

[59] Shigekawa T, Ijichi N, Takayama S, Tsuda H, Ikeda K, Horie K, Osaki A, Saeki T, Inoue S (2009). FOXP1 as a Potential ER Coregulator in Human Breast Cancer. Cancer Res.

[60] van Boxtel R, Gomez-Puerto C, Mokry M, Eijkelenboom A, van der Vos KE, Nieuwenhuis EE, Burgering BM, Lam EW, Coffer PJ (2013). FOXP1 acts through a negative feedback loop to suppress FOXO-induced apoptosis. Cell Death Differ.

[61] Gabut M, Samavarchi-Tehrani P, Wang X, Slobodeniuc V, O’Hanlon D, Sung HK, Alvarez M, Talukder S, Pan Q, Mazzoni EO, Nedelec S, Wichterle H, Woltjen K, Hughes TR, Zandstra PW, Nagy A, Wrana JL, Blencowe BJ (2011). An alternative splicing switch regulates embryonic stem cell pluripotency and reprogramming. Cell.

[62] Quesada V, Conde L, Villamor N, Ordonez GR, Jares P, Bassaganyas L, Ramsay AJ, Bea S, Pinyol M, Martinez-Trillos A, Lopez-Guerra M, Colomer D, Navarro A, Baumann T, Aymerich M, Rozman M, Delgado J, Gine E, Hernandez JM, Gonzalez-Diaz M, Puente DA, Velasco G, Freije JM, Tubio JM, Royo R, Gelpi JL, Orozco M, Pisano DG, Zamora J, Vazquez M (2012). Exome sequencing identifies recurrent mutations of the splicing factor SF3B1 gene in chronic lymphocytic leukemia. Nat Genet.

[63] Pajer P, Pecenka V, Kralova J, Karafiat V, Prukova D, Zemanova Z, Kodet R, Dvorak M (2006). Identification of potential human oncogenes by mapping the common viral integration sites in avian nephroblastoma. Cancer Res.

[64] Datta J, Kutay H, Nasser MW, Nuovo GJ, Wang B, Majumder S, Liu CG, Volinia S, Croce CM, Schmittgen TD, Ghoshal K, Jacob ST (2008). Methylation mediated silencing of MicroRNA-1 gene and its role in hepatocellular carcinogenesis. Cancer Res.

[65] Rao DS, O’Connell RM, Chaudhuri AA, Garcia-Flores Y, Geiger TL, Baltimore D (2010). MicroRNA-34a perturbs B lymphocyte development by repressing the forkhead box transcription factor Foxp1. Immunity.

[66] Streubel B, Vinatzer U, Lamprecht A, Raderer M, Chott A (2005). T(3;14) (p14.1;q32) involving IGH and FOXP1 is a novel recurrent chromosomal aberration in MALT lymphoma. Leukemia.

[CR67] The pre-publication history for this paper can be accessed here: http://www.biomedcentral.com/1471-2407/14/840/prepub

